# HMG‐CoA Synthase‐2 Deficiency: Neonatal Hyperammonemic Coma and Abnormal Metabolic Screening Resembling Maple Syrup Urine Disease

**DOI:** 10.1002/jmd2.70028

**Published:** 2025-06-22

**Authors:** Hathaipat Vaseenon, Thipwimol Tim‐Aroon, Vitchayaporn Emarach Saengow, Areeporn Sangcakul, Parith Wongkittichote, Arthaporn Khongkraparn, Duangrurdee Wattanasirichaigoon

**Affiliations:** ^1^ Division of Medical Genetics, Department of Pediatrics, Faculty of Medicine Ramathibodi Hospital Mahidol University Bangkok Thailand; ^2^ Department of Pediatrics, Faculty of Medicine Vajira Hospital Navamindradhiraj University Bangkok Thailand; ^3^ Department of Pediatrics Maharat Nakhon Ratchasima Hospital Nakhon Ratchasima Thailand; ^4^ Department of Pathology, Faculty of Medicine Ramathibodi Hospital Mahidol University Bangkok Thailand

**Keywords:** HMGCS2, hyperammonemia, hypoglycemia, metabolic acidosis

## Abstract

Mitochondrial HMG‐CoA synthase‐2 (HMGCS2) deficiency is characterized by hypoketotic hypoglycemia, metabolic acidosis, hepatomegaly, and encephalopathy with onset between 3 and 36 months of age. Approximately 50 cases were reported worldwide. We describe two patients with HMGCS2 deficiency. Patient 1 presented on day of life 7 with a sepsis‐like condition, coma, metabolic acidosis, and marked elevation of ammonium level at 1081 μmol/L. Metabolic screening demonstrated elevated valine and leucine/isoleucine concentrations, resembling maple syrup urine disease (MSUD). The patient received a blood exchange transfusion, which lowered the ammonium level to 92 μmol/L. Urine organic acid analysis did not confirm MSUD. At 1 year and 4 months, the patient experienced acute decompensation again. Exome sequencing revealed a homozygous *HMGCS2* variant, c.1502G>C (p.Arg501Pro). Patient 2, an older sibling of Patient 1, was healthy but diagnosed through genetic testing. Both patients exhibited abnormal biochemical profiles, including dicarboxylic aciduria and increased urinary excretion of 4‐hydroxy‐6‐methyl‐2‐pyrone (4‐HMP) after 8 h of fasting, suggesting that a clinically asymptomatic patient, like Patient 2, may eventually develop acute decompensation. Therefore, preemptive treatment with fasting avoidance with or without l‐carnitine during intercurrent illness should be advised. The present cases were the second and third patients of p.Arg501Pro homozygosity. The elevated branched‐chain amino acids in the metabolic screening (without including alloisoleucine) and the described organic acid profile can be found during the catabolic state, resembling MSUD, and severe hyperammonemia is an uncommon phenotype and an exception to neonatal decompensation in HMGCS2 deficiency. Our findings demonstrated intrafamilial variability and expanded the clinical and biochemical spectrum of HMGCS2 deficiency.

**Summary**

- Neonatal decompensation in HMGCS2 deficiency can result in severe hyperammonemia and abnormal metabolic screening resembling MSUD.

**Abbreviations**
CPS1: carbamoyl phosphate synthase 1
DOL: day of life
FAOD: fatty acid oxidation disorder
HMGCS2: mitochondrial HMG‐CoA synthase‐2
LCAD: long‐chain acyl‐CoA dehydrogenase
MCAD: medium‐chain acyl‐CoA dehydrogenase
MSUD: maple syrup urine disease
OTC: ornithine transcarbamoylase (OTC)
PAA: plasma amino acid
TMS: tandem mass spectrometry
UOA: urine organic acid
VLCAD: very long‐chain acyl‐CoA dehydrogenase
WES: whole‐exome sequencing

## 1 Introduction

Mitochondrial HMG‐CoA synthase‐2 (HMGCS2), encoded by the gene *HMGCS2* (OMIM #600234), located on chromosome 1p12, is an enzyme responsible for the first reaction of ketogenesis, specifically the condensation of acetoacetyl‐CoA and acetyl‐CoA to form HMG‐CoA [1]. Pathogenic variants of *HMGCS2* have been associated with a rare autosomal recessive disorder of ketogenesis, namely HMGCS2 deficiency (OMIM#605911), which is characterized by hypoketotic hypoglycemia, metabolic acidosis, hepatomegaly, and encephalopathy, usually precipitated by intercurrent illness and/or prolonged fasting [1]. To date, approximately 50 cases have been reported worldwide. The onset of HMGCS2 deficiency usually occurs between 3 and 36 months of age [2, 3, 4, 5, 6]. Only one case of neonatal onset presenting with severe hyperammonemia and coma has recently been described in a Vietnamese cohort of HMGCS2 deficiency [7].

Here, we present two siblings affected by HMGCS2 deficiency, including the younger sibling who presented with a sepsis‐like condition, neonatal hyperammonemic coma, and an abnormal metabolic screening suggestive of maple syrup urine disease (MSUD); the older sibling was diagnosed through genetic testing of family members. Our findings demonstrated intrafamilial variability and expanded the phenotypic and biochemical spectrum of HMGCS2 deficiency.

## 2 Case Presentation

### 2.1 Patient 1

The patient was a 38‐week female newborn with a birth weight of 2480 g (third centile), delivered after an uneventful pregnancy. On the day of life (DOL) 7, the patient had watery diarrhea, low‐grade fever, poor sucking, and rapid deterioration to difficulty breathing (respiratory rate of 68 breaths per minute, BPM), mottling skin, and was unresponsive to stimuli on the following day. At the emergency room of a local hospital, the patient was found comatose, with hypotension, desaturation (pulse oximetry of 85%), and hypoglycemia with blood glucose of 1.1 mmol/L (normal: 3.9–5.5 mmol/L). The patient was given three bolus doses of 25% dextrose intravenously (2 mL/kg), followed by continuing fluid resuscitation, endotracheal intubation, and ventilatory support. Her body weight was measured at 2220 g, or 10% weight loss compared to birth weight. There was borderline hepatomegaly with a liver span of 7 cm, no abnormal breath sounds heard, and no abnormal odor.

Laboratory testing showed severe wide anion gap metabolic acidosis, with the following results: pH < 6.8, serum bicarbonate (CO_2_) 3.3 mmol/L, calculated anion gap 38 (normal: 8–16 mmol/L), and elevated BUN and creatinine levels at 32.7 mmol/L and 84.8 mmol/L, respectively. Serum calcium and phosphate were within normal limits, 2.15 mmol/L (normal: 2.0–2.5 mmol/L) and 2.4 mmol/L (normal: 1.4–3.0 mmol/L). There was an elevated lactate level at 4.1 mmol/L (normal: 0.5–2.2 mmol/L), hyperammonemia at 1081 μmol/L (normal: < 50 μmol/L), mild elevation of AST at 245 U/L (normal: 32–162 U/L), normal ALT level at 38 U/L (normal: 5–33 U/L), and a low serum ketone level (beta‐hydroxybutyric acid or BHB) at 0.2 mmol/L (normal: < 0.6 mmol/L). Urinalysis was within normal limits, including a pH of 5.5, specific gravity of 1.015, and negative for ketones, sugar, and protein. Complete blood count showed anemia (Hct 29.8%), leukocytosis with mild neutrophilia (Wbc 21 400; neutrophils 76% and lymphocytes 10%), and a low normal platelet count at 119 × 10^9^/L (normal: 140–400 × 10^9^/L). Examination of cerebrospinal fluid (CSF) revealed normal findings. Blood, urine, and CSF cultures were negative. The patient developed focal‐clonic seizures. Empirical antibiotics and phenobarbital were given. A CT/MRI scan of the brain and an electroencephalogram (EEG) were not done.

Since newborn screening for inborn metabolic disorders was not available, a dried blood specimen for comprehensive metabolic screening using tandem mass spectrometry (TMS; acylcarnitine included) was collected on DOL7, when the patient was symptomatic. The metabolic screening, which included acylcarnitine profile, had a rapid turnaround time of 2 days, compared to 5–14 days of plasma amino acid (PAA) and urine organic acid (UOA) analyses.

The acylcarnitine profile revealed markedly elevated branched‐chain‐amino acid levels: Leu\Ile\Pro‐OH at 1121 μmol/L (normal: 69–218 μmol/L) and valine 893 μmol/L (normal: 66–218 μmol/L), suggesting maple syrup urine disease (MSUD). The alloisoleucine level was not available on the testing panel. Given the high suspicion of MSUD, the patient underwent one cycle of total blood exchange (2× blood volume) due to the unavailability of dialysis at the local hospital. Metabolic management included withholding protein, administering a high‐caloric glucose‐electrolyte infusion, and providing sodium benzoate and l‐carnitine for suspected organic acidemia and MSUD. Plasma ammonium level at 6 h after the blood exchange was normal at 92 μmol/L, and 10 days later, it was 61 μmol/L. The patient was extubated on DOL11 and fed via a nasogastric tube.

The patient was referred to our center on DOL18, or 11 days after symptom onset, while she was drowsy but had been spontaneously breathing, with oxygen supplementation and mild subcostal retraction. The patient received a metabolic formula with limited branched‐chain amino acid (BCAAs) intake, l‐carnitine, sodium benzoate, and thiamine. The results of the pre‐exchange PAA profile showed an elevation of multiple amino acid levels: leucine 479 μmol/L (normal: 42–133 μmol/L); valine 529 μmol/L (normal: 74–273 μmol/L); isoleucine 169 μmol/L (normal: 15–75 μmol/L); glycine 721 μmol/L (normal: 94–462 μmol/L); lysine 1305 μmol/L (normal: 25–225 μmol/L), which was consistent with a catabolic state in the setting of severe illness. Of note, plasma alloisoleucine was not measured due to unavailability at that time. The UOA profile demonstrated increased excretion of 2‐hydroxy‐isovaleric acid, 3‐hydroxy‐isovaleric acid, and 2‐hydroxy‐3‐methylvaleric acid, which may indicate MSUD or a non‐specific catabolic state. Given the absence of 2‐hydroxyisocaproic acid and 2‐ketoisocaproic acid in the urine and the profiles of PAAs, MSUD was unlikely. The metabolic formula was gradually discontinued and replaced with a regular infant formula with a gradual increase in whole protein intake and close monitoring for metabolic derangement. The patient completely gained consciousness on DOL26, and she had a full oral feeding. Repeat ammonium levels, PAA, and UOA on DOL20, 29, and 41 were nondiagnostic for an inborn metabolic disorder. The patient was discharged on DOL60 on breast milk and bottle feeding. She had regular follow‐up appointments every 2–4 months, during which her developmental milestones were appropriate, and her ammonium levels were within normal range.

At the age of 1 year and 4 months, the patient presented at a local hospital with altered consciousness following acute gastroenteritis (3 times of vomiting and 2 times of diarrhea). She was found to have metabolic acidosis (serum bicarbonate 14 mmol/L), mild hyperammonemia (63 μmol/L), and low blood glucose (3.6 mmol/L). After hydration with intravenous fluids containing high dextrose, the symptoms improved, and she returned to her baseline after 2 weeks of admission. However, critical specimens were not collected during this time due to the lack of communication. The metabolic tests, including PAA, UOA, and acylcarnitine profile, performed at 1 year and 8 months (not taking carnitine), while the patient was well, were unremarkable. Given that fatty acid oxidation disorder (FAOD) could not be excluded due to the history of recurrent hypoglycemia and encephalopathy, the patient was instructed to take L‐carnitine and avoid fasting. Although we were aware that a FAOD was very unlikely with a completely normal acylcarnitine profile, and that carnitine supplementation could be harmful in some FAODs, such as very‐long‐chain acyl‐CoA dehydrogenase (VLCAD) deficiency, the absence of cardiomegaly/cardiac dysfunction did not support VLCAD; after balancing the risk and benefit, we decided to prescribe a carnitine supplement.

At 2 years and 8 months, whole‐exome sequencing (WES) was conducted, using Illumina NovaSeq 6000 (Macrogen, South Korea) with Agilent's SureSelect (V5 and 6G) for target enrichment and 150 bp Pair End mode (~average read depth 125×). The exome analysis pipeline, following Broad Institute's best practice guidelines for GATK v3.4 (https://www. broadinstitute.org/) and the established protocols, including the interpretation of the variants, was employed [8, 9]. The exome analysis did not reveal candidate variants for the following human phenotype ontology: hyperammonemia (HP:0001987), metabolic acidosis (HP:0001942), and MSUD‐causative genes [10]. Moreover, genes underlying FAOD and carnitine cycle disorders, including medium‐chain acyl‐CoA dehydrogenase (MCAD: *ACADM* gene), long‐chain acyl‐CoA dehydrogenase (LCAD: *ACADL* gene), and very long‐chain acyl‐CoA dehydrogenase (VLCAD: *ACADVL* gene), Carnitine palmitoyltransferase I and II (*CPT1A* and *CPT2* genes), and systemic primary carnitine (*SLC22A5* gene) deficiencies were included in the analysis using the human phenotype ontology aforementioned, but did not reveal candidate variants. The carnitine supplement was continued for 2 years before stopping. A reanalysis 3 years later with an additional phenotype, hypoglycemia (HP:0001943), revealed a homozygous pathogenic variant in the *HMGCS2* gene, designated as c.1502G>C (p.Arg501Pro). Sanger sequencing confirmed homozygosity of the variant in Patient 1 and one of her older siblings (Patient 2). Both parents were heterozygous for this variant.

To investigate the biochemical abnormalities that might have been missed in the past, the patient was asked to do a safe fast for 8 h and then collected blood and urine samples for an array of tests, including UOA, PAA, dried blood spots for acylcarnitine profile (not under carnitine treatment), serum ammonia, serum lactate, blood glucose, and creatine kinase (CK). The UOA analysis revealed massive dicarboxylic aciduria (adipic acid and suberic acid) as well as 4‐hydroxy‐6‐methyl‐2‐pyrone (4‐HMP), consistent with HMGCS2 deficiency, and the acylcarnitine profile demonstrated elevations of C8, C10, C12, C12:1, C14:1, and C14:2 with C2/C0 ratio less than 3 (normal: < 3), and low normal C0 level at 17.1 μmol/L (normal: 14.1–65.5 μmol/L). The full acylcarnitine panel results are detailed in Table S1. Her PAA and CK levels were within normal limits (CK 26 U/L, normal: 50–231 U/L), as well as the remaining test results.

As for the laboratory methods, urine organic acid analysis was conducted using ethyl acetate/diethyl ether extraction, as described in previously published protocols [11]. The presence of 4‐hydroxy‐6‐methyl‐2‐pyrone in the urine was confirmed by comparison to a pure reference standard (MilliPoreSigma, Burlington, MA). Acylcarnitine profiling was performed using flow injection analysis–tandem mass spectrometry of underivatized acylcarnitines, following established protocols [12].

### 2.2 Patient 2

Patient 2, a 13‐year‐old brother of Patient 1, was reportedly healthy and normally developed and had no history of metabolic decompensation throughout his childhood. Physical examination was unremarkable. To determine if he might have the subclinical manifestation of HMGCS2 deficiency, the patient was instructed to fast for 8 h. Samples were then collected to perform the aforementioned array of tests, as done in Patient 1. His UOA profiles showed moderate excretion of urinary dicarboxylic acids and mild excretion of 4‐HMP, and his acylcarnitine profiles revealed elevations of C8, C10, C12:1, and C14:1 with a C2/C0 ratio less than 3 (normal: < 3), and a low normal C0 level at 19.6 μmol/L. There was a slight increase in CK level at 509 U/L, and normal findings of the other laboratory tests.

## 3 Discussion

We describe the second case of neonatal‐onset HMGCS2 deficiency, presenting with severe metabolic acidosis and hyperammonemic coma in the setting of preceding diarrhea. Initial metabolic profiles raised a suspicion of MSUD. This patient represented the youngest symptomatic patient with HMGCS2 deficiency ever reported. We also identified a clinically asymptomatic patient who exhibited biochemical abnormalities of HMGCS2 deficiency after a period of fasting.

HMGCS2 is a key enzyme in the mitochondrial ketogenesis pathway, responsible for converting acetyl‐CoA and acetoacetyl‐CoA into HMG‐CoA, a precursor of ketone bodies. The marked hyperammonemia in the neonate (Patient 1) was intriguing. Although hyperammonemia is not a major manifestation of HMGCS2 deficiency, mild elevation of blood ammonia has been noted [2, 7]. Severe neonatal hyperammonemia of 983 μmol/L has been reported in one case in a Vietnamese cohort [7]. The pathophysiology of hyperammonemia in HMGCS2 deficiency remains poorly understood. It is hypothesized that impaired ketogenesis and increased glucose consumption may result in augmented protein catabolism to support both ketogenesis and gluconeogenesis, as evidenced by the elevation of multiple amino acids in Patient 1. Deamination of these amino acids excessively produces ammonia, which may contribute to the development of hyperammonemia [13]. Furthermore, the enzymes involved in the urea cycle may be dysregulated in HMGCS2 deficiency. Studies have demonstrated that acetylation and deacetylation of carbamoyl phosphate synthase 1 (CPS1) and ornithine transcarbamoylase (OTC) play crucial roles in regulating their activity [14, 15]. Increased mitochondrial acetyl‐CoA levels in HMGCS2 deficiency may promote protein acetylation and reduce the activity of these enzymes. Notably, research in mice has shown that neonatal ketogenesis plays a critical role in preventing mitochondrial protein hyperacetylation and preserving mitochondrial bioenergetics, as *Hmgcs2* knockout mice exhibit inadequate energy production and mitochondrial protein hyperacetylation [16]. The protein responsible for deacetylating CPS1 is Sirtuin 5 (SIRT5), an NAD^+^‐dependent enzyme [15]. Given the elevated levels of acetyl‐CoA, the metabolic flux likely shifts into the tricarboxylic acid (TCA) cycle, leading to increased production of NADH. The resulting imbalance between NAD and NADH may lead to the inactivation of SIRT5, thereby impairing its ability to deacetylate CPS1 and ultimately disrupting the urea cycle. Taken together, these factors may have contributed to the development of hyperammonemia in patients with HMGCS2 deficiency.

The observed elevations in multiple acylcarnitines are most likely attributable to the fasting state and/or during the metabolic decompensation. Notably, both probands did not show elevated levels of acetylcarnitine (C2) or 3‐hydroxybutyrylcarnitine (C4OH) in the specimens obtained after 8 h of fasting. Instead, their free carnitine (C0) levels were in the low‐normal range, and the C2/C0 ratio was normal, similar to many individuals with HMGCS2 deficiency previously reported [7]. It is well established that elevations in long‐chain acylcarnitines, especially in the context of low or inappropriately normal C2, can suggest defects in fatty acid oxidation. In the context of HMGCS2 deficiency, we propose that the increased long‐chain acylcarnitines reflect an enhanced flux through fatty acid oxidation pathways as part of the metabolic response to fasting. However, due to impaired ketogenesis, free carnitine may become increasingly sequestered as acylcarnitines, contributing to the observed reduction in C0. As noted in a previous study, an elevated C2/C0 ratio greater than three has been suggested as indicative of HMGCS2 deficiency. It is important to note that most biochemical profiles in the literature have been obtained either during acute metabolic decompensation or in the well state. Consequently, data describing the acylcarnitine profile during fasting in the absence of decompensation are limited. Therefore, the diagnostic utility of this pattern remains unclear.

The elevations in C8, C10, C12, and C12:1 acylcarnitine species can also be observed in medium‐chain acyl‐CoA dehydrogenase (MCAD) deficiency; however, the level of C10:1, considered a hallmark biomarker of MCAD deficiency, was within the normal range in Patients 1 and 2. Additionally, the UOA analysis did not reveal the presence of suberylglycine or hexanoylglycine, both of which are established biomarkers for MCAD deficiency. Taken together, these findings did not support a diagnosis of MCAD deficiency in both patients. Instead, the multiple elevations of these acylcarnitine species likely reflected physiological changes in fatty acid metabolism during the fasting state.

In retrospect, the diarrhea in Patient 1 led to major stress and hypoglycemia, which consequently pressured ketogenesis and gluconeogenesis. The wide gap and severe metabolic acidosis could be attributed to sepsis, poor tissue perfusion, and defective HMGCS2 enzymes. The elevated branched‐chain amino acids might not be simply explained by the result of severe illness alone, but also by the body's impaired ketogenesis that led to excessive protein breakdown. As for the differential diagnosis of MSUD, ketonuria and elevated serum uric acid concentrations are known to correlate with high leucine levels and can be used as a predictor of acute decompensation of MSUD [17]. However, uric acid level was not measured in Patient 1, and we might have missed the fact of absent ketonuria as a clue against MSUD.

To date, approximately 50 pathogenic or likely pathogenic variants in *HMGCS2* have been reported [1, 7]. Among these, two‐thirds are null alleles (stop‐gain, splice site, frameshift, deletion/duplication), and the remaining are missense variants [1, 7]. The c.1502G>C (p.Arg501Pro) variant has been reported four times, mostly in the compound heterozygous state with another premature truncation mutation [18, 19, 20], and in a homozygous state once in a Chinese patient [18]. The severity of the metabolic phenotype in patients with the homozygous p.Arg501Pro variant is expected to be significant but less severe compared to compound heterozygous variants involving p.Arg501Pro and a premature truncation. A functional study of the p.Arg501Pro variant demonstrated that, although the variant produced protein at a level comparable to the wild‐type, as measured by Western blot analysis, its enzymatic activity was completely abolished [21].

Additional learning points from the present cases were as follows: First, the delayed definite diagnosis of Patient 1 was due to the *HMGCS2* not being included in the Human Phenotype Ontology (HPO) terms for metabolic acidosis and hyperammonemia. We hope that the present case could urge the addition of phenotypes of hyperammonemia and metabolic acidosis to this gene. Second, the inclusion of phenotypes as many as possible in the analysis would increase the yield of diagnosis; in this case, although hypoglycemia is a main finding in HMGCS2 we did not include hypoglycemia in the initial analysis, given it was relatively nonspecific and related to poor feeding, and we simply focused on the more prominent abnormality (metabolic acidosis and hyperammonemia). Third, reanalysis of undiagnosed cases is always helpful because of an expanding list of new phenotypes linked to the known disorders and new causative genes identified. It has been shown that reanalysis every 1–2 years could result in 10%–15% incremental diagnosis [22, 23]. Last, close monitoring and preemptive treatment of cases with suspected inborn metabolic disorders are necessary for keeping the patient safe whilst searching for a definite diagnosis is ongoing, like in Patient 1 who received treatment for FAOD and related disorders prior to the definite diagnosis.

Intrafamilial variability has been reported in various inborn errors of metabolism, including HMGCS2 deficiency. Our patients are the second and third patients homozygous for the p.Arg501Pro variant [18]. In our study, the two patients exhibited significant differences in severity; the younger sister became severely symptomatic following a gastroenteritis, while the 13‐year‐old brother has remained asymptomatic so far. Interestingly, abnormal UOAs were observed in both patients, including 4‐HMP and dicarboxylic aciduria in the context of low ketones. Previous studies have demonstrated that 4‐HMP can serve as a reliable marker of HMGCS2 deficiency, being detected in many patients during decompensation [1, 24, 25]. However, urine samples must be collected during acute decompensation, as the level of 4‐HMP may normalize during the healthy state. Following the fasting, we were able to detect 4‐HMP, even in Patient 2, who appeared clinically asymptomatic. This finding confirms an abnormal biochemical process in a clinically asymptomatic patient, suggesting that such individuals may eventually develop acute decompensation when facing major stress (such as severe illness, accidents, and military training). Therefore, we advised Patient 2 to avoid fasting, similar to Patient 1. It is also of interest to explore the potential effect of other variants and the epigenetic landscape, which may exert protective or deleterious effects in HMGCS2 deficiency, contributing to phenotypic variability despite similar underlying biochemical abnormalities.

HMGCS2 deficiency generally has a good prognosis, with treatment focused on fasting avoidance and ensuring adequate caloric intake during intercurrent illness. Taking carnitine during intercurrent illness has been practiced in some centers. Despite a good prognosis, a hidden patient could be at risk of acute encephalopathy, which could result in permanent neurological sequelae or death if not diagnosed and properly treated in a timely fashion. Identification of hidden patients in affected families can also prevent unexpected death due to acute metabolic decompensation.

## 4 Conclusion

The elevated branched‐chain amino acids in the metabolic screening (without including alloisoleucine) and the described organic acid profile can be found during the catabolic state, resembling MSUD, and severe hyperammonemia is an uncommon phenotype and an exception to neonatal decompensation in HMGCS2 deficiency.

## Author Contributions

H.V. and D.W. conceptualized and designed the study, writing the manuscript draft. H.V., V.E.S., and D.W. provided patient care and collected and analyzed clinical data. A.S. and P.W. performed and/or analyzed the biochemical studies. P.W. wrote part of the manuscript. H.V. and A.K. performed and analyzed molecular data. T.T.‐A. supervised molecular analysis. D.W. and P.W. acquired funding. D.W. supervised the study and performed critical editing. All authors have read and agreed to the published version of the manuscript.

## Consent

Yes. The study was approved by Ramathibodi Hospital Human Research Ethics Committee (COA.MU2024/839).

## Conflicts of Interest

The authors declare no conflicts of interest.

## Supporting information


**Table S1.** Acylcarnitine profile of two patients after 8‐h fast.

## Data Availability

All data generated and analyzed in this study are presented in this published article.
