# Supplementary material for: HMG‐CoA Synthase‐2 Deficiency: Neonatal Hyperammonemic Coma and Abnormal Metabolic Screening Resembling Maple Syrup Urine Disease
Source: JIMD Rep. 2025 Jun 22;66(4):e70028. doi: 10.1002/jmd2.70028 (PMC12182750; doi:10.1002/jmd2.70028)
Supplement: Supplementary file 1 — Table S1. Acylcarnitine profile of two patients after 8‐h fast. [file JMD2-66-e70028-s001.docx]

**Supplementary data**

**HMG-CoA synthase-2 deficiency: Neonatal hyperammonemic coma and abnormal metabolic screening resembling maple syrup urine disease**

Hathaipat Vaseenon^1,2^, Thipwimol Tim-Aroon^1^, Vitchayaporn Emarach Saengow^3^, Areeporn Sangcakul^4^, Parith Wongkittichote^1^, Arthaporn Khongkraparn^1^, Duangrurdee Wattanasirichaigoon^1,^*

**Table S1** Acylcarnitine profile of 2 patients after 8-hr fast

| Acylcarnitine profile | **Patient 1:** age 7 yr, | **Patient 2:** age 13 yr, | Reference range  for age >5 day (nmol/ml) |
| --- | --- | --- | --- |
| C0 | 17.10 | 19.60 | 14.152 - 65.520 |
| C2 | 12.60 | 6.99 | 2.430 – 23.348 |
| C3 | 1.09 | 0.72 | 0.298 – 2.705 |
| C3DC \ C4OH | 0.08 | 0.07 | 0.030 – 0.180 |
| C4 | 0.24 | 0.17 | 0.080 – 0.342 |
| C4DC \ C5OH | 0.43 | **0.59 H** | 0.120 – 0.435 |
| C5 | 0.09 | 0.15 | 0.060 – 0.685 |
| C5:1 | 0.01 | 0.01 | 0.000 – 0.010 |
| C5DC \ C6OH | 0.05 | 0.04 | 0.020 – 0.102 |
| C6 | 0.06 | 0.06 | 0.020 – 0.080 |
| C6DC | 0.03 | 0.03 | 0.020 – 0.102 |
| C8 | **0.10 H** | **0.12 H** | 0.020 – 0.090 |
| C8:1 | 0.03 | 0.02 | 0.010 – 0.160 |
| C10 | **0.17 H** | **0.21 H** | 0.020 – 0.120 |
| C10:1 | 0.06 | 0.07 | 0.010 – 0.070 |
| C10:2 | 0.01 | 0.01 | 0.000 – 0.020 |
| C12 | **0.11 H** | 0.09 | 0.010 – 0.100 |
| C12:1 | **0.10 H** | **0.10 H** | 0.010 – 0.050 |
| C14 | 0.12 | 0.09 | 0.030 – 0.280 |
| C14:1 | **0.13 H** | **0.11 H** | 0.020 – 0.090 |
| C14:2 | **0.04 H** | 0.03 | 0.010 – 0.030 |
| C14OH | 0.01 | 0.01 | 0.000 – 0.020 |
| C16 | 1.51 | 0.86 | 0.388 – 4.391 |
| C16:1 | 0.12 | 0.08 | 0.020 – 0.192 |
| C16OH | 0.01 | 0.01 | 0.010 – 0.030 |
| C16:1OH | 0.03 | 0.02 | 0.010 – 0.050 |
| C18 | 0.81 | 0.44 | 0.160 – 1.602 |
| C18:1 | 1.40 | 0.84 | 0.368 – 2.487 |
| C18:2 | 0.26 | 0.12 | 0.060 – 0.570 |
| C18OH | 0.01 | 0.00 | 0.000 – 0.020 |
| C18:1OH | 0.02 | 0.02 | 0.010 – 0.050 |
